# Yangyin Runchang Decoction Improves Intestinal Motility in Mice with Atropine/Diphenoxylate-Induced Slow-Transit Constipation

**DOI:** 10.1155/2017/4249016

**Published:** 2017-12-18

**Authors:** Feng Jiang, Jin-Yong Zhou, Jian Wu, Fang Tian, Xuan-Xuan Zhu, Chang-Le Zhu, Bo-Lin Yang, Yu-Gen Chen

**Affiliations:** ^1^No. 1 Clinical Medical College, Nanjing University of Chinese Medicine, Nanjing, Jiangsu 210023, China; ^2^Jiangsu Province Hospital of TCM, The Affiliated Hospital of Nanjing University of TCM, Nanjing 210029, China

## Abstract

This study assessed the efficacy and mechanism of action of Yangyin Runchang decoction (YRD) in the treatment of slow-transit constipation (STC). ICR mice were randomly divided into four groups (*n* = 10/group) and treated with saline (normal control; NC), atropine/diphenoxylate (model control; MC; 20 mg/kg), or atropine/diphenoxylate plus low-dose YRD (L-YRD; 29.6 g/kg) or high-dose YRD (H-YRD; 59.2 g/kg). Intestinal motility was assessed by evaluating feces and the intestinal transit rate (ITR). The serum level of stem cell factor (SCF) and changes in interstitial cells of Cajal (ICCs) were also evaluated. Additionally, the expression of SCF and c-kit and the intracellular Ca^2+^ concentration [Ca^2+^]_*I*_ were investigated. Fecal volume and ITR were greater in the L-YRD and H-YRD groups than in the MC group. The serum SCF level was lower in the MC group than in the NC group; this effect was ameliorated in the YRD-treated mice. Additionally, YRD-treated mice had more ICCs and elevated expression of c-kit and membrane-bound SCF, and YRD also increased [Ca^2+^]_*I*_* in vitro* in isolated ICCs. YRD treatment in this STC mouse model was effective, possibly via the restoration of the SCF/c-kit pathway, increase in the ICC count, and enhancement of ICC function by increasing [Ca^2+^]_*i*_.

## 1. Introduction

Constipation is a frequently encountered disorder in clinical practice [1]. The global incidence of chronic constipation is approximately 14%. The slow-transit constipation (STC) subtype occurs relatively frequently [2]. Prucalopride is a new 5-hydroxytryptamine receptor 4 agonist that is beneficial for treating chronic simple laxative-resistant constipation; however, there is little evidence of its beneficial effect in STC management [3]. Additionally, a highly variable success rate (39–100%) for surgical treatment of STC has been reported in the literature [4]. Therefore, it is important to find complementary therapies for this condition.

STC is characterized by an increased colonic transit time, as measured using radionucleotides or radiopaque markers. The etiology of STC has not been elucidated; however, several studies have shown that a decrease in the number of interstitial cells of Cajal (ICCs) may play an important role in the pathophysiology of this condition [5–7]. The stem cell factor (SCF)/c-kit pathway is important for the development and maintenance of the ICC phenotype [8, 9]. When it is blocked, the number of ICCs may be reduced in the intestine, which can result in STC. In this case, ICC function can be restored by activating the SCF/c-kit pathway [10], and this may provide an approach to the treatment of STC.

Some herbal medicines have been shown to be effective treatments for constipation [11]. In particular, Yangyin Runchang decoction (YRD) produces marked effects and has been used clinically to treat STC for nearly 60 years by the famous Chinese medicine professor Zhu Bingyi. YRD is composed of Zengye soup and Zhizhu pills, which are important decoctions for the treatment of constipation in traditional Chinese medicine.

In a previous study, we found that the expression of c-kit mRNA in rats with STC increased after treatment with YRD. We also found that the effect of YRD decreased when it was used at a dose lower than the clinical dose. Therefore, we hypothesized that YRD may increase the number of ICCs via activation of the SCF/c-kit pathway. In the present study, we investigated the effects of YRD on the expression of SCF and c-kit in a mouse model of STC. We also assessed the serum SCF level. Finally, we investigated the potential mechanism underlying these effects of YRD.

## 2. Materials and Methods

### 2.1. Animals

Forty ICR mice (weight, 27 ± 2 g) were purchased from the Experimental Animal Center of Nantong University (Jiangsu, China) and maintained at the Animal Center of Jiangsu Traditional Chinese Medicine Hospital (Jiangsu, China) after adaptation for 1 week. The mice were kept in a room under a 12/12-h day/night cycle. All mice were treated in accordance with the Guidelines for Care and Use of Laboratory Animals of Nanjing University of Traditional Chinese Medicine (Nanjing, China).

### 2.2. Drugs and Reagents

All herbs were sourced from Jiangsu Province Hospital of Traditional Chinese Medicine (Nanjing, China). YRD was prepared as follows: a mixture of herbs (195 g) comprising 20 g Huomaren, 20 g Yuliren, 20 g Shengdihuang, 20 g Gualouren, 20 g Maidong, 20 g Xuanshen, 30 g Shengbaizhu, 15 g Zhike, 15 g Shengma, and 15 g Liushenqu was boiled in 1800 ml of distilled water, refluxed, and extracted. A 3 g/ml herbal water extract was prepared from the mixed decoctions. The extract was stored at −20°C until use. Atropine/diphenoxylate was purchased from Changzhou Kangpu Pharmaceutical Co., Ltd. (Lot number: 1701023; Changzhou, China).

### 2.3. Experimental Design

Atropine/diphenoxylate (0.025 mg atropine and 2.5 mg diphenoxylate in one tablet) was used to induce constipation in the mice, as previously reported [12, 13]. The mice were randomly divided into the following study groups: the normal control group (NC) was treated with normal saline (0.2 ml/10 g); the model control group (MC) was treated with atropine/diphenoxylate (20 mg/kg); the low-dose YRD group (L-YRD) was treated with atropine/diphenoxylate and 1.48 g/ml YRD (0.2 ml/10 g); the high-dose YRD group (H-YRD) was treated with atropine/diphenoxylate and 2.96 g/ml YRD (0.2 ml/10 g). In accordance with the equivalent human dose, mice were administered 9.1-fold the clinical adult dosage [14]; the L-YRD dose was 29.6 g/kg/d, which was equivalent to the human clinical dose, while the H-YRD group received twice this dosage.

Atropine/diphenoxylate was administered once daily for 15 consecutive days, whereas YRD was administered twice daily in days 16–30 in the L-YRD and H-YRD groups. All drugs were administered via gastric gavage.

### 2.4. Fecal Volume and Water Content

Total 24-h feces were collected and weighed once weekly. Fresh feces were weighed and recorded as wet weight (*A*). The dry weight (*B*) was obtained after drying fresh feces in an oven for 3 h. The percentage of water in the feces was calculated as (*A* − *B*)/*A* × 100%.

### 2.5. Propulsion of Activated Carbon in the Intestines

All mice were fasted for 18 h, after which they were administered 0.5 ml of 5% activated carbon by oral gavage. Thirty minutes later, the mice were sacrificed by cervical dislocation. Next, the pylorus to the rectum was removed from the end of the intestinal tract. The length of the intestine and the length of the activated carbon in the intestine were measured in a relaxed state. Intestinal transit rate (ITR) was the length from the pylorus to the end of the intestine stained with activated carbon, expressed as a percentage of the total length of the intestine.

### 2.6. Immunohistochemical Analysis

Paraffin-embedded colon tissues were cut into 3-*μ*m-thick sections. After deparaffinization, the sections were subjected to antigen recovery, blocked, and then incubated with rabbit anti-c-kit (1 : 50) and anti-SCF (1 : 500) antibodies at 4°C overnight. Diaminobenzidine was used to detect the immunocomplex, whereas hematoxylin was used for nuclear counterstaining. An immunoglobulin-negative control was used to eliminate nonspecific binding. The sections were examined by light microscopy. Images were analyzed using Image Pro Plus 6.0 (Media Cybernetics, Inc., Rockville, MD, USA). The integrated optical density (IOD) from three fields of each slice was calculated using Intel IPP software (version 6.0; Intel, Mountain View, CA, USA) and used to represent protein expression [15].

### 2.7. Assay of Blood SCF Levels

Blood samples were collected and centrifuged at 3000 rpm for 20 min to obtain serum. SCF levels were determined using an enzyme-linked immunosorbent assay kit (RayBiotech, Inc., Norcross, GA, USA).

### 2.8. Reverse Transcription Polymerase Chain Reaction

Colon tissues were homogenized with Trizol (Life Technologies Corporation, Grand Island, NY, USA). Total RNA (500 ng) was reverse-transcribed into cDNA using PrimeScript RT Master Mix (TaKaRa Biotechnology, Dalian, China). The procedures were performed strictly in accordance with the manufacturers' protocols. Amplification of cDNA using the primers listed in Table 1 was performed by an ABI 7500 system (Applied Biosystems, Foster City, CA, USA) using the DNA-binding dye, SYBR Green. Glyceraldehyde 3-phosphate dehydrogenase (GAPDH) was used as an internal reference gene and the ΔΔCt method was used to assess variation in c-kit and SCF mRNA expression.

### 2.9. Western Blot Analysis

Colon tissue samples were homogenized and lysed in sodium dodecyl sulfate (SDS) polyacrylamide gel electrophoresis sample buffer. The mixture was boiled and centrifuged at 12,000 rpm for 20 min, after which the supernatant was collected. Equal amounts of protein (30 *μ*g) from each sample were separated on 10% SDS polyacrylamide gels, transferred onto polyvinylidene difluoride membranes (Millipore, Billerica, MA, USA), and incubated with 5% bovine serum albumin for 1 h. The membranes were probed with a c-kit monoclonal antibody (K45; Thermo Fisher Scientific, Waltham, MA, USA) and an anti-SCF antibody (Abcam, Cambridge, UK) at 4°C overnight. Next, the membranes were washed and incubated with horseradish-peroxidase-linked secondary antibodies. An enhanced chemiluminescence kit (Millipore) was then used to detect the immunoreactive signals.

### 2.10. ICC Purification and Identification

ICCs were purified and identified according to the method described by Gong et al. [16]. The intestines of Balb/c mice (approximately one week old) were removed and incubated in a solution containing collagenase (1.3 mg/ml; Sigma, St. Louis, MO, USA), trypsin inhibitor (2 mg/ml), and adenosine triphosphate (0.27 mg/ml). Single-cell suspensions were incubated with antibodies as follows: ICCs were labeled with anti-mouse CD117 (c-kit) PE (clone 2B8; 0.125 *μ*g/10^7^ cells in 100 *μ*l of M199; eBioscience, Inc., San Diego, CA, USA); mast cells and other leukocytes were identified using the PerCP anti-mouse CD45 (clone 30-F11; 0.25 *μ*g/10^6^ cells in 100 *μ*l of M199; BioLegend, San Diego, CA, USA); macrophages were incubated with FITC anti-mouse F4/80 (clone BM8; 0.25 *μ*g/10^6^ cells in 100 *μ*l of M199; BioLegend). Cells were analyzed by fluorescence-activated cell sorting (FACS) on a BD FACSAria III (BD Biosciences, San Jose, CA, USA). First, forward scatter and side scatter gates were set to select cells with the light scattering properties of live cells. Second, F4/80^−^ and CD45^−^ cell gates were set to exclude macrophages, mast cells, and other leukocytes. Next, ICCs (c-kit^+^) were separated from F4/80^−^ CD45^−^ cells. Purified ICCs were plated on glass-bottomed dishes (NEST Biotechnology Co., Ltd., Wuxi, China) and incubated with M199 (Gibco, Grand Island, NY, USA) supplemented with 1% penicillin-streptomycin solution (Gibco) and 10% fetal bovine serum (Gibco). After 3 days, cultured ICCs were fixed with 4% paraformaldehyde and incubated with anti-mouse CD117 (c-kit) PE (0.25 *μ*g/ml) and anti-TMEM16A antibody (Anoctamin1, ANO1; 2 *μ*g/ml; Abcam) at 4°C overnight. The cells were then rinsed with phosphate-buffered saline, incubated with AF 488 goat anti-rabbit antibody (Fcmacs, Nanjing, China) for 1 h, and observed by confocal laser scanning microscopy (CLSM) using an LSM710 microscope (Carl Zeiss, Oberkochen, Germany).

### 2.11. Measurement of Intracellular Ca^2+^ Concentration [Ca^2+^]_*i*_

Cultured ICCs were incubated in M199 containing 5 *μ*M Fluo-3/AM (Beyotime, Nanjing, China) in a 95% O_2_/5% CO_2_ incubator for 1 h, rinsed twice with D-Hank's solution, and observed by CLSM (LSM710). The basal and maximum fluorescence levels after treatment with different concentrations of YRD were recorded. The mean fluorescence intensity was used to represent the [Ca^2+^]_*i*_.

### 2.12. Statistical Analysis

Data were analyzed with SPSS 22.0 software (IBM Corporation, Armonk, NY, USA). The results have been presented as the mean ± standard deviation. Significant differences in the results between groups were analyzed by one-way analysis of variance followed by least significance difference test. *P* < 0.05 was considered statistically significant.

## 3. Results

### 3.1. Changes in Feces

**Figure 1:**
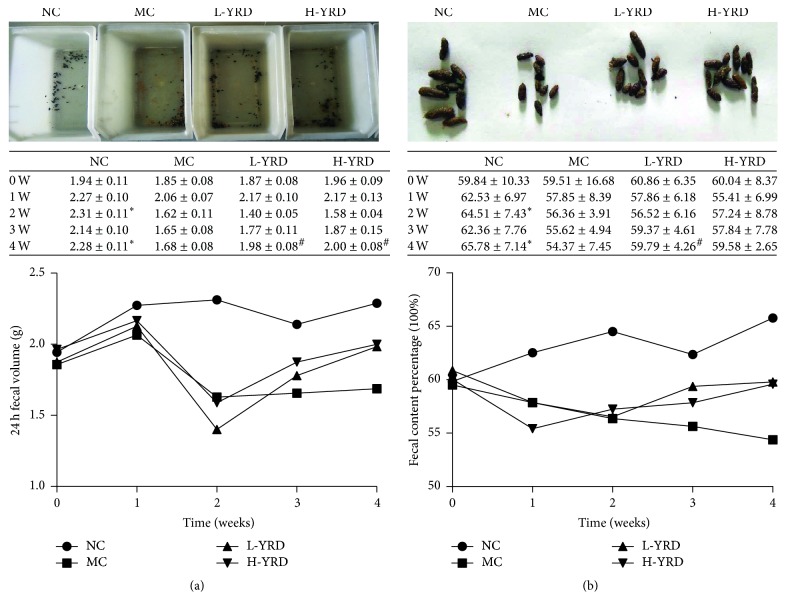
Effects of YRD on the feces of mice with atropine/diphenoxylate-induced STC. (a) Twenty-four-hour fecal volumes declined significantly over 2 weeks in the MC group (*∗* indicates  *P* < 0.01); however, at 4 weeks, the 24-h fecal volumes in the L-YRD and H-YRD groups were higher than that of the MC group (# indicates  *P* < 0.01). (b) The water content in the feces of mice in the MC group was significantly reduced (*∗* indicates  *P* < 0.05 when compared to the NC group). In mice treated with YRD, the fecal water content was significantly higher, especially in the L-YRD group (# indicates *P* < 0.05 when compared to the MC group).

The volume of feces collected over 24 h decreased significantly in the MC group (*P* < 0.01); however, it increased in the L-YRD and H-YRD groups (*P* < 0.01) (Figure 1(a)). Mice in the MC group had drier and smaller feces than those in the NC group. In addition, the amount of water in the feces was lower in the MC group than in the NC group (*P* < 0.05); however, mice treated with YRD showed higher fecal water levels, especially in the L-YRD group (*P* < 0.05) (Figure 1(b)).

### 3.2. Effect of YRD on ITR

**Figure 2:**
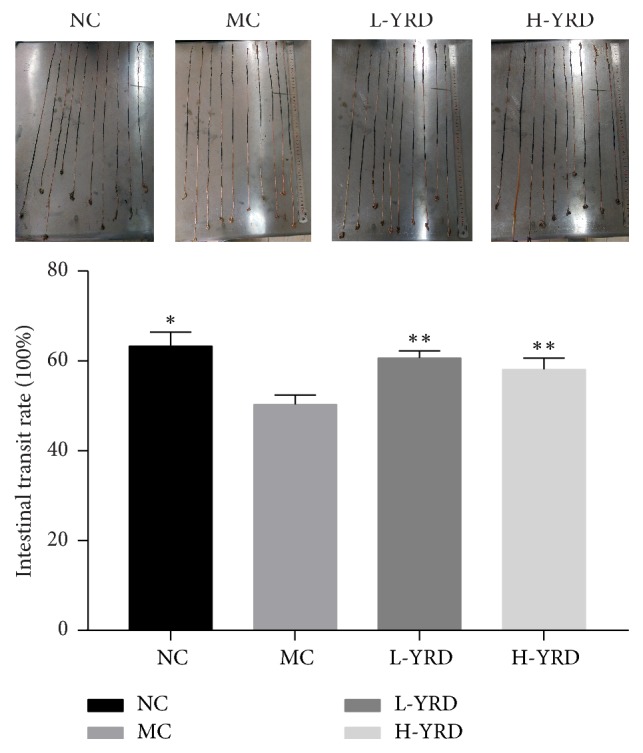
Effects of YRD on ITR in mice with atropine/diphenoxylate-induced STC. ITR was slower in the MC group than it was in the NC group (*∗* indicates  *P* < 0.01); however, it was higher in the L-YRD and H-YRD groups than it was in the MC group (*∗∗* indicates  *P* < 0.05).

ITR was significantly lower in the MC group than in the NC group (50.28 ± 2.17% versus 63.32 ± 3.09%; *P* < 0.01). However, ITR was higher in the L-YRD (60.62 ± 1.64%) and H-YRD (58.09 ± 2.57%) groups than in the MC group (*P* < 0.05). The difference in ITR between the L-YRD and H-YRD groups was not statistically significant (Figure 2).

### 3.3. Changes in the ICCs

**Figure 3:**
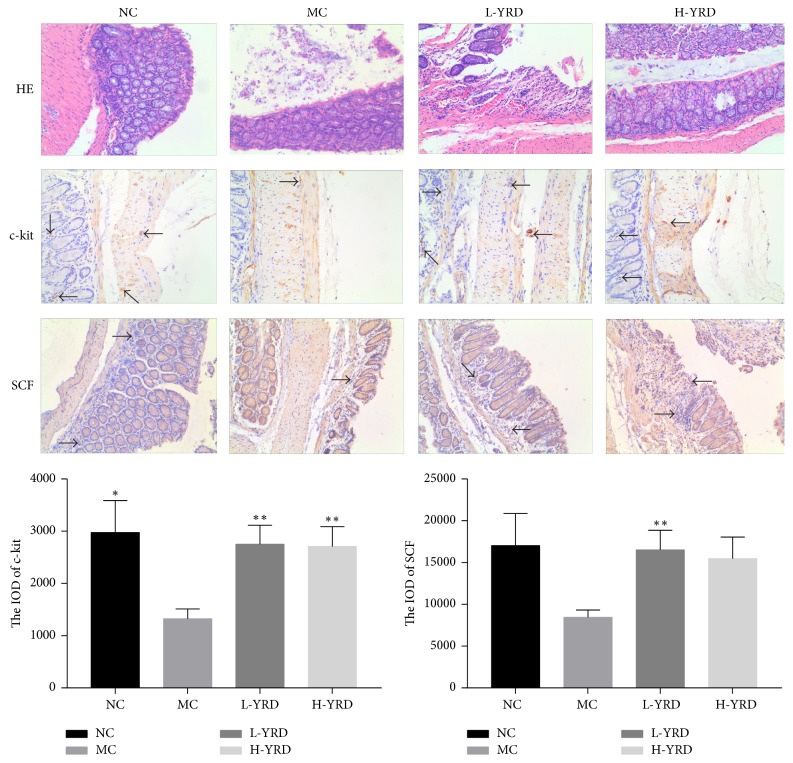
Pathological changes in the mice. The histopathological analysis identified mucosal damage and inflammatory cell infiltration into the mucosa in the MC group; however, these changes were ameliorated in the L-YRD and H-YRD groups. SCF levels and the number of c-kit-positive cells were lower in the MC group than they were in the NC group; however, they were preserved in the YRD treatment groups. Arrows indicate the positive cells. The IOD of c-kit was lower in the MC group than it was in the NC group (*∗* indicates* P* < 0.01); however, it was higher in the L-YRD and H-YRD groups than it was in the MC group (*∗∗* indicates* P* < 0.05). The IOD of SCF was lower in the MC group than it was in the NC group, and it was higher in the L-YRD group than it was in the MC group (*∗∗* indicates* P* < 0.05).

Partial mucosal damage and inflammatory cell infiltration into the mucosa were observed in the constipated mice. However, mucosal damage was alleviated in the YRD-treated mice. The number of ICCs in the intestines was estimated immunohistochemically. Many ICCs were found in the submucosa; additionally, some were in the myenteric plexus and intramuscular space, forming a small network. The results were analyzed by two pathologists. Fewer c-kit-positive cells were present in the MC group than in the NC group (Figure 3). More c-kit-positive cells were observed in the L-YRD and H-YRD groups than in the MC group. The IOD for c-kit in the MC group was lower than that of the NC group (1326.34 ± 186.05 versus 2974.51 ± 613.39; *P* < 0.01). In addition, the c-kit IOD values were significantly higher in the L-YRD (2750.61 ± 364.57) and H-YRD (2709.66 ± 379.45) groups than in the MC group (*P* < 0.05 for both comparisons).

### 3.4. Effect of YRD on the SCF/c-Kit Pathway

**Figure 4:**
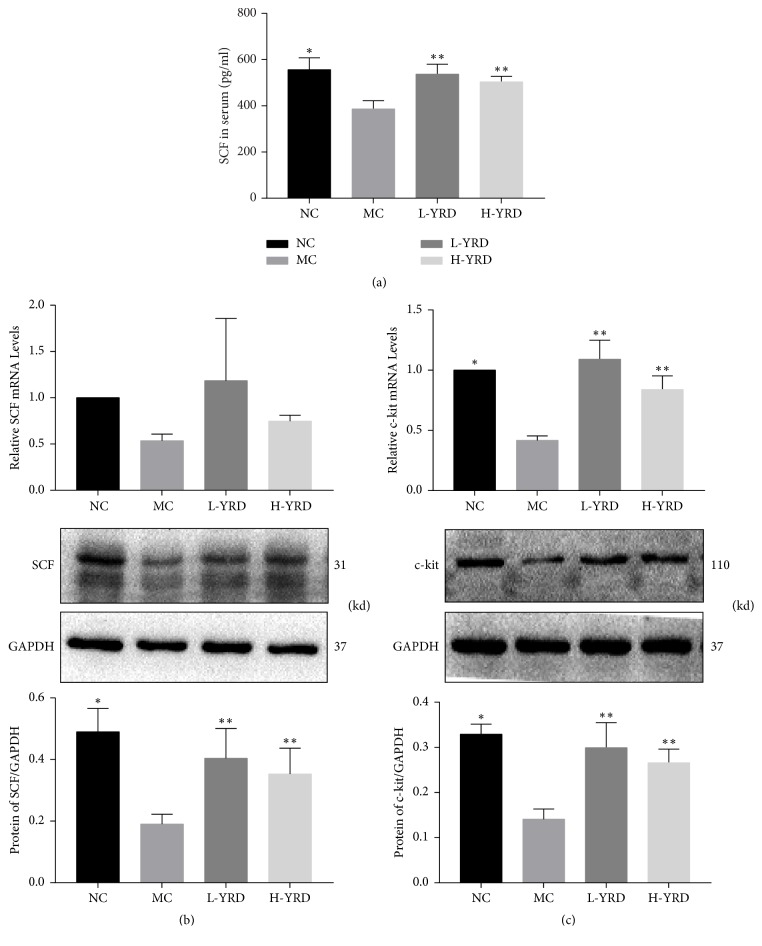
Effects of YRD on SCF and c-kit expression in mice with atropine/diphenoxylate-induced STC. (a) A lower serum SCF level was observed in the MC group (*∗* indicates  *P* < 0.01 when compared to the NC group). After treatment with YRD for 2 weeks, higher serum levels of SCF were detected in both the L-YRD and H-YRD groups (*∗∗* indicates  *P* < 0.05). (b) A lower protein expression of SCF was observed in the MC group (*∗* indicates  *P* < 0.01 when compared to the NC group), and this change was ameliorated in both the L-YRD and H-YRD groups. Similar results were obtained for SCF mRNA expression. (c) A lower protein expression of c-kit was observed in the MC group (*∗* indicates  *P* < 0.05 when compared to the NC group); however, this change was ameliorated in the L-YRD and H-YRD groups (*∗∗* indicates  *P* < 0.05). Similar results were obtained for c-kit mRNA expression.

The serum level of SCF was significantly lower in the MC group than in the NC group (386.58 ± 36.02 pg/ml versus 556.16 ± 51.62 pg/ml; *P* < 0.01). In addition, significant (*P* < 0.01) attenuation of this atropine/diphenoxylate-induced decrease in serum SCF was observed in both the L-YRD (537.20 ± 42.88 pg/ml) and H-YRD (503.44 ± 23.94 pg/ml) groups. There was no significant difference between the serum SCF levels of the L-YRD and H-YRD groups (Figure 4(a)). Similarly, SCF protein and mRNA expression levels were significantly lower in the MC group than in the NC group; however, they were significantly (*P* < 0.05) higher in the L-YRD and H-YRD groups than in the MC group. There were no statistically significant differences between the SCF protein and mRNA levels in the L-YRD and H-YRD groups (Figure 4(b)).

C-kit protein and mRNA expression of colon tissue in the MC group was lower than that in the NC group (*P* < 0.05). The levels of c-kit protein and mRNA were elevated in both the L-YRD and H-YRD groups (*P* < 0.05), with no statistically significant difference between the YRD-treated groups (Figure 4(c)).

### 3.5. Purification and Identification of ICC by FACS and CLSM

**Figure 5:**
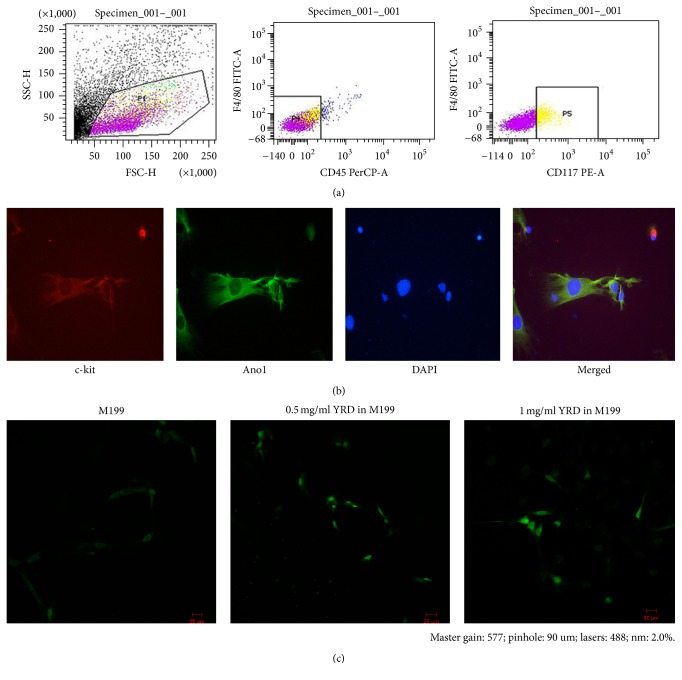
[Ca^2+^]_*i*_ in ICCs. (a) ICCs were purified by FACS and accounted for approximately 5.7% of the total cell number. (b) ICCs were identified by immunofluorescent c-kit and ANO1 staining using CLSM. (c) The [Ca^2+^]_*i*_ fluorescent signal was low in ICCs cultured in normal M199; however, it increased significantly (*P* < 0.01) in those cultured in M199 containing YRD. There was a statistically significant difference between the [Ca^2+^]_*i*_ of the ICCs exposed to L-YRD and H-YRD (*P* < 0.01).

F4/80^−^ CD45^−^ cells accounted for approximately 37.8%, and c-kit^+^ cells accounted for approximately 5.7% of the total cell number (Figure 5(a)). We observed that the cells were triangular, round, or spindle-shaped, with two to five long processes and large nuclei. In addition, adjacent cells that expressed c-kit and ANO1 proteins formed a network. Isolated cells that were double-stained were identified as ICCs (Figure 5(b)).

### 3.6. Effect of YRD on [Ca^2+^]_*i*_ in Purified ICCs

The basal fluorescence level of ICCs in D-Hank's solution was approximately 8.31 ± 1.45; however, this was slightly elevated in normal M199 (13.25 ± 2.86), representing the [Ca^2+^]_*i*_ level. The maximum [Ca^2+^]_*i*_ fluorescence levels observed in cells cultured in M199 containing 0.5 mg/ml YRD (32.22 ± 3.47) or 1 mg/ml YRD (45.05 ± 2.32) were significantly higher than those observed in cells cultured in normal M199 (*P* < 0.01 for both comparisons; Figure 5(c)).

## 4. Discussion

Chronic constipation is a common clinical complaint. STC is a motility disorder characterized by a marked increase in the total ITR [17]. In the present study, the ITR was significantly slower in mice treated with atropine/diphenoxylate than in normal mice, showing that STC had been successfully induced. In addition, the ITR increased in YRD-treated groups. This indicates that YRD increases intestinal motility and may therefore provide an effective therapy for STC.

In the clinic, inflammation may be involved in the development of STC. Li et al. [18] used the iTRAQ labeling technique coupled with two-dimensional liquid chromatography-tandem mass spectrometry to analyze colon tissue from 43 patients with STC, and identified inflammatory cell infiltration. Villanacci et al. [19] found that patients with intractable STC had a significantly greater number of colonic lymphoid aggregates than those in the control specimens. The number of colonic mast cells, a type of inflammatory cell, was shown to be elevated in severely constipated patients [20]. In the present study, we observed minor mucosal damage and inflammatory cell infiltration into the mucosa in the mice model, but the characteristics of dry stool and slow transit of colon featured by the mice model were consistent with the definition of STC by the American Gastroenterology Association [21]. This indicated that atropine/diphenoxylate-induced constipation provided a suitable mouse model of STC.

Although the pathogenesis of STC is unclear, a reduction in the ICC number is an important feature of the condition [22]. ICCs are the pacemakers of the gastrointestinal tract, as they generate slow wave currents that trigger rhythmic phasic contractions [23–25]. Maintenance of ICCs requires SCF/c-kit signaling [8], which suggests that repressing this pathway can result in a decrease in the number of ICCs. This may lead to the development of many diseases such as acute cholecystitis [26], cholesterol gallstones [27], gastric dysmotility [28], diabetes-related voiding dysfunction [29], and diabetic gastroparesis [30]. However, there are no reports of investigations into whether the reduced ICC number in STC patients is associated with an inhibition of the SCF/c-kit signaling pathway. SCF is an important ligand for kit. It has two isoforms, namely soluble SCF (sSCF) and membrane-bound SCF (mSCF), which are both important for ICC function; however, mSCF produces a more sustained effect on ICCs [31]. In addition to increasing the number of ICCs [29], sSCF can partially reverse pathological ICC changes in diabetic mice [32]. In our investigation, we found that the levels of SCF in serum (sSCF) and colon tissue (mSCF) were lower in the MC group than in the other study groups. The numbers of c-kit-positive cells and the levels of c-kit protein expression were also lower in the MC group. However, sSCF and mSCF levels were restored in the mice treated with YRD. The numbers of c-kit-positive cells and levels of c-kit protein expression were also restored by YRD treatment, with no significant difference in these results between the L-YRD and H-YRD groups. This indicated that it may not be necessary to increase the dose of YRD to improve its efficacy. Consequently, we hypothesized that the SCF/c-kit pathway was impaired due to a decreased expression of SCF. This leads to a decrease in ICC number, which may contribute to the etiology of STC. YRD provided an effective treatment for the mouse STC model, probably because it increased the number of ICCs by restoring the SCF/c-kit pathway. Other studies have shown that changes in SCF and c-kit expression are consistent with recovery of the SCF/c-kit pathway and could increase the number of ICCs [28, 33].

FACS is the main method used to purify ICCs. In the present study, ICCs were purified by FACS and accounted for about 5.7% of the total cell number. This result was similar to that obtained by Gong et al. [16]. Furthermore, ICCs were identified by immunofluorescent double staining. ANO1 is a new and highly selective molecular marker for ICCs [34]. The cultured cells were positive for both c-kit and ANO1, indicating that they were ICCs.

Oscillation of the [Ca^2+^]_*i*_ in ICCs plays a key role in generating slow waves, which induce spontaneous contractions of smooth muscles. The uptake and periodic release of Ca^2+^ from intracellular stores that are operated by the inositol 1,4,5-trisphosphate receptor appear to be the main processes involved in [Ca^2+^]_*i*_ oscillation, which drives the initiation of pacemaker currents [35]. Torihashi et al. [36] observed slow periodic contractions that were associated with [Ca^2+^]_*i*_ oscillations in ICC clusters using Ca^2+^ imaging [37]. In the present study, continuous observation of fluorescence by CLSM did not detect the significant spontaneous [Ca^2+^]_*i*_ signals in cultured ICCs that were previously noted by Gong et al. [16]. This may reflect weakening of the spontaneous [Ca^2+^]_*i*_ signals over extended cells culture periods. A separate study also observed few rhythmic spontaneous [Ca^2+^]_*i*_ signals in ICCs [37]. When cultured ICCs were incubated in M199 containing YRD, their fluorescence increased significantly, indicating that [Ca^2+^]_*i*_ fluxes may be strengthened by YRD. However, the specific mechanism underlying this increase in [Ca^2+^]_*i*_ remains to be elucidated.

## 5. Conclusions

The ICC number and function decreased in the MC group, mimicking a change that may be involved in the pathogenesis of STC. The potential therapeutic mechanism of YRD may involve restoring the SCF/c-kit pathway, increasing the number of ICCs, and enhancing their function by increasing [Ca^2+^]_*i*_.

## Figures and Tables

**Table 1:** Primer nucleotide sequences.

Gene	Primers	Nucleotide sequences 5′-3′
c-kit	Forward	AACGATGTGGGCAAGAGTTC
	Reverse	CCTCGACAACCTTCCATTGT
SCF	Forward	ACGTGGACCAGTGGAAGAAC
	Reverse	TTGCACATTCAGCATTCCTC
GAPDH	Forward	CACCCCATTTGATGTTAGTG
	Reverse	CCATTTGCAGTGGCAAAG
